# Changing Epidemiological Patterns of Infection and Mortality Due to Hepatitis C Virus in Poland

**DOI:** 10.3390/jcm12123922

**Published:** 2023-06-08

**Authors:** Agnieszka Genowska, Dorota Zarębska-Michaluk, Birute Strukcinskiene, Arturas Razbadauskas, Anna Moniuszko-Malinowska, Jonas Jurgaitis, Robert Flisiak

**Affiliations:** 1 Department of Public Health, Medical University of Bialystok, 15-295 Bialystok, Poland; 2 Department of Infectious Diseases and Allergology, Jan Kochanowski University, 25-317 Kielce, Poland; dorota1010@tlen.pl; 3 Faculty of Health Sciences, Klaipeda University, LT-92294 Klaipeda, Lithuania; birute.strukcinskiene@ku.lt (B.S.); rektorius@ku.lt (A.R.); jonas.jurgaitis@ku.lt (J.J.); 4 Department of Infectious Diseases and Neuroinfections, Medical University of Bialystok, 15-540 Bialystok, Poland; anna.moniuszko-malinowska@umb.edu.pl; 5 Department of Infectious Diseases and Hepatology, Medical University of Bialystok, 15-540 Bialystok, Poland

**Keywords:** hepatitis, HCV, diagnosis, mortality, trend, COVID-19 impact, rural, urban, gender, age

## Abstract

Introduction: Limited information is available on trends in hepatitis C virus (HCV) infection, particularly in Central Europe. To address this knowledge gap, we analyzed HCV epidemiology in Poland, considering socio-demographic characteristics, changing patterns over time, and the impact of the COVID-19 pandemic. Material and Methods: We examined HCV cases (diagnosis and deaths) reported by national registries and used joinpoint analysis to estimate time trajectories. Results: Between 2009 and 2021, there were changes in the trends of HCV, shifting from positive to negative in Poland. Among men, there was a significant increase initially in diagnosis rate of HCV in rural areas (annual percent change, APC_2009–2016_ +11.50%) and urban areas (APC_2009–2016_ +11.44%) by 2016. In subsequent years until 2019, the trend changed direction, but the reduction was weak (*P*_trend_ > 0.05) in rural areas (−8.66%) and urban areas (−13.63%). During the COVID-19 pandemic, the diagnosis rate of HCV dramatically decreased in rural areas (APC_2019–2021_ −41.47%) and urban areas (APC_2019–2021_ −40.88%). Among women, changes in the diagnosis rate of HCV were less pronounced. In rural areas, there was a significant increase (APC_2009–2015_ +20.53%) followed by no significant change, whereas changes occurred later in urban areas (APC_2017–2021_ −33.58%). Trend changes in total mortality due to HCV were mainly among men, with a significant decrease in rural (−17.17%) and urban (−21.55%) areas from 2014/2015. Conclusions: The COVID-19 pandemic reduced HCV diagnosis rates in Poland, especially for diagnosed cases. However, further monitoring of HCV trends is necessary, along with national screening programs and improved linkage to care.

## 1. Introduction

Hepatitis C virus (HCV) infection is a major public health problem that results in high morbidity and mortality rates worldwide, along with significant economic costs [1,2,3]. It is estimated that 58 million people are living with chronic HCV infection, and mortality among those diagnosed with HCV is higher than in the general population [4,5,6,7]. However, only 20% of people living with HCV know about their disease, and a mere 8% of patients diagnosed with HCV infection have received antiviral therapy [8]. The transmission of HCV mainly occurs through injecting drugs, unsafe healthcare practices, blood transfusions without screening, and sexual intercourse in patients with coexisting sexually transmitted diseases [4,6,9]. In 55–85% of patients, acute hepatitis C progresses to chronic hepatitis C, which can later cause serious and even fatal complications [4]. Patients with chronic HCV infection are at increased risk of liver fibrosis, leading to cirrhosis with a risk of decompensation, and hepatocellular carcinoma. This ultimately results in reduced quality of life and shortened lifespan [6,10]. In 2019, hepatitis C caused 15.3 million global disability-adjusted life years (DALYs) and 0.6% of all global DALYs. Acute hepatitis, cirrhosis, and liver cancer contributed to 1.7, 79.5, and 18.9% of DALYs due to hepatitis C, respectively [11]. Therefore, HCV has become a major health threat across the European region [12].

In the European area, the prevalence of HCV infection ranges from 0.1 to 5.9%, with considerable variation between country-specific rates [12,13]. These variations are attributed to differences in the availability of treatment and national policies governing its administration. Information about trends in HCV infection is scarce, especially in Central Europe. Despite the fact that the Central European countries, including Poland, do not have problems with access to diagnostics and treatment, there is still a poor linkage to care due to insufficient healthcare staff, and a major gap in viral hepatitis care in this region. However, none of the Central European countries has a chance to meet the goals set by the WHO to eliminate viral hepatitis from the list of public threats by 2030, unless barriers such as lack of political will and lack of screening programs are removed soon. The introduction of screening tests in general practitioners’ offices gives some hope, but these activities are carried out on a small scale. In addition, the COVID-19 pandemic has significantly reduced the chances of achieving the goals set by the WHO [14,15,16,17].

In Poland, one of the biggest countries in Central Europe with a population of about 38 million, the prevalence of HCV in the adult population is estimated at 0.9–1.9% for the period 2009–2020. During the same period, active HCV infection diagnosed based on the presence of HCV-RNA was 0.4–0.6% of respondents, corresponding to approximately 140,000–200,000 infected people on the national scale [15,18,19]. Since 2015, highly effective therapeutic regimens containing direct-acting antiviral (DAA) drugs have been available in Poland, and that is not without impact on the number of actively infected patients. However, according to the WHO program, the lack of national screening programs in Poland complicates the achievement of HCV elimination [16]. To achieve the goal of eliminating HCV as defined by WHO, a country needs to have robust epidemiological information to plan and monitor effective prevention and control programs [20]. Some data on epidemiological patterns in Central Europe, particularly in Poland, are available, but unfortunately, no long-term analysis after the COVID-19 pandemic, showing the detection of infections and death trends due to HCV infection, has been conducted so far [18,21].

The aim of the study was to analyze infection and mortality due to HCV in Poland by socio-demographic characteristics (gender, age, and place of residence), considering changing patterns over time, and the impact of COVID-19 pandemic.

## 2. Materials and Methods

### 2.1. Study Design

In this nationwide population-based retrospective study in Poland, we analyzed all registered cases of HCV infection and deaths due to HCV from 2009 to 2021. The study included anonymous information on diagnosed cases of HCV infection and deaths, along with socio-demographic characteristics such as gender, age, and place of residence. No informed consent was required for our epidemiological analysis since, in compliance with Polish law, all cases of infectious diseases are registered and reported for epidemiological surveillance. HCV infection is a reportable disease or cause of death, and thus all recorded cases of HCV during the analyzed 13-year period were included in our study. Due to the nature of the study, which involved an analysis of secondary epidemiological data, the approval of the local Bioethics Committee was not required.

### 2.2. Definition of HCV

In Poland, records of newly diagnosed HCV cases were based on the national case definition, which underwent changes during the analyzed period of 2009–2019 [22]. The definition adopted in 2009, in line with the criteria of epidemiological surveillance unified in the European Union (EU) by the Decision 2008/426/EU, recorded all laboratory-confirmed cases, regardless of the clinical picture (detection of anti-HCV antibodies, confirmed by another test for the presence of antibodies or detection of HCV nucleic acid in the blood serum). For comparability of data over time, cases of hepatitis C from 2009 to 2013 were classified based on both the 2005 and 2009 definitions [22].

In 2014, the definition was modified in accordance with the EU decision (2012/506/EU), and in 2015, cases were recorded simultaneously based on both the 2005 and 2014 definitions, covering all laboratory-confirmed cases (demonstration of the presence of anti-HCV antibodies confirmed by the antibody confirmatory test, detection of HCV nucleic acid or core antigen, regardless of the clinical picture) [22]. In 2018, another modification was adopted based on the EU decision (2018/945/EU), and in 2019, laboratory criteria were divided into acute, chronic, or unspecified forms.

From 2019, in Poland, the definition of an acute case is confirmed if it meets the EU criteria or has a symptomatic case of HCV with icterus or elevated transaminase activity (≥350 IU/mL or ALT ≥ 10 times the upper limit of the norm). The chronic form is confirmed by the detection of HCV RNA or HCV-core antigen, or the presence of anti-HCV antibodies confirmed by a test in persons older than 18 months without evidence of fighting the infection. The unspecified form is registered if it does not meet the criteria of acute or chronic form [22]. In addition to physician diagnosis, positive HCV test results have also been reported by laboratories since 2014.

### 2.3. Data Sources and HCV Variables

Diagnosed cases of HCV in the Polish population are recorded in a register maintained by the National Institute of Public Health–National Research Institute (NIPH–NRI), and publicly archived database Epimeld [23]. The register includes cases of HCV infection confirmed in medical institutions and recorded by the local State Sanitary Inspection. Data from the Central Statistical Office on registered HCV deaths were also used.

The study included diagnosed new cases of infection and deaths due to HCV, identified using the International Statistical Classification of Diseases and Related Health Problems, Tenth Revision (ICD-10), codes for acute HCV (B17.1), chronic HCV (B18.2), mixed infections HBV and HCV (B16.0–16.9, B18.0–18.1), and deaths due to HCV (B17.1, B18.2). The diagnosis rate of HCV was calculated as the number of new cases per 100,000 population for each calendar year, and was stratified by socio-demographic characteristics such as gender, age groups (≤14, 15–24, 25–34, 35–44, 45–54, 55–64, ≥65), and place of residence (rural and urban areas). Data on deaths due to HCV were used for the analysis of mortality. The ASMR was calculated using the direct standardization method and the 2013 edition of the European Standard Population as the reference population [24]. ASMR rates per 100,000 population were determined for each calendar year, taking into account gender, age groups (≤44, 45–64, and ≥65), and place of residence.

### 2.4. Statistical Analysis

Categorical variables were presented as proportions and compared using a chi-square test. Two-proportion tests with Bonferroni adjustment for multiple comparisons were also implemented to compare HCV cases (diagnosis and death) between individual pairs of subgroups. Wilcoxon’s signed-rank tests were used to compare the distributions of HCV diagnosis and mortality rates between rural and urban populations.

Joinpoint regression analysis was applied to examine changes associated with HCV rates of diagnosis and mortality trends during the last 13 years. Joinpoint tests of significance use a Monte Carlo permutation method and utilized generalized linear models assuming a Poisson distribution [25]. This analysis allowed us to identify years when a significant change in the linear slope of the trend (on a log scale) was detected during the study period. In regression models, the segmented lines were joined at points called joinpoints, and each joinpoint showed a significant change in the slope.

For our analysis of HCV, we started with the minimum number of joinpoints (e.g., 0 joinpoints, which is a straight line) and tested a maximum of two joinpoints (corresponding to three-line segments) that were significantly different in each model. The results of the estimated linear trends of HCV with zero joinpoints were presented as average annual percentage change (AAPC). The estimated linear trends with one or two joinpoints were presented as partial annual percentage changes (APC) [26,27]. The differences in examined trends of HCV between rural and urban areas were also examined using the Wald test [25]. IBM^®^ SPSS^®^ Statistics for Windows, Version 24.0-IBM Corp., Armonk, NY, USA, was used for all statistical analyzes. A *p*-value lower than 0.05 was considered statistically significant in all analyzes.

## 3. Results

Between 2009 and 2021 in Poland, a total of 36,069 new cases of HCV were diagnosed, with 0.9% of these cases involving a combination of HCV and HBV infections. A higher proportion of newly diagnosed HCV cases were found in urban areas (73.9%) compared to rural areas (26.1%). Differences in new HCV cases by place of residence were observed in both genders, with a statistically significant difference (*p* ≤ 0.001). Among women, the highest proportion of new HCV cases was in the age group 55–64 in both urban (25.0%) and rural areas (22.1%). For men in urban areas, the highest proportion of new HCV cases was in the age group 35–44 (23.3%), while in rural areas it was in the age group 45–54 (21.1%). The smallest proportion of new HCV cases was found in the youngest age group for both genders, with 0.5 and 0.9% in urban and rural areas for men, and 0.6 and 0.9% in urban and rural areas for women, respectively.

A total of 2101 deaths due to HCV were recorded between 2009 and 2021, with a higher proportion of these cases in urban areas (76.4% or 1605 deaths) compared to rural areas. In both urban and rural areas, deaths due to HCV were more frequently observed in men (54.4 and 59.5%, respectively) than in women (45.6 and 40.5%, respectively). Of the total deaths due to HCV, a significant proportion were due to chronic HCV (97.2% or 2043 deaths). In both urban and rural areas, deaths due to chronic HCV were more frequent in men (54.6 and 59.9%, respectively) than in women (45.4 and 40.1%, respectively). Acute HCV deaths were recorded more frequently in men (60.0 and 53.8% in urban and rural areas, respectively) compared to women (40.0 and 46.2% in urban and rural areas, respectively).

Table 1 presents the descriptive statistics for the distribution of HCV diagnosis rates and mortality rates. The median HCV diagnosis rate was found to be twice as high in urban areas compared to rural areas. The median diagnosis rates were significantly different between urban and rural populations for all age groups and genders, except for the youngest age group (≤14 years). In men, the total rates of HCV in urban areas were 9.29 per 10^5^ compared to 4.34 per 10^5^ in rural areas (*p* ≤ 0.001), and in women, they were 7.98 per 10^5^ vs. 4.70 per 10^5^ (*p* ≤ 0.001), respectively. The lowest diagnosis rates of HCV were observed in the age group ≤ 14 years, both in urban and rural areas, in men (0.24 per 10^5^ vs. 0.23 per 10^5^, respectively), and in women (0.37 per 10^5^ vs. 0.16 per 10^5^, respectively). The highest diagnosis rates of HCV were found in men aged 45–54 years, both in urban and rural areas (14.54 per 10^5^ vs. 7.37 per 10^5^, *p* ≤ 0.001), and in women aged 55–64 years (13.86 per 10^5^ vs. 9.87 per 10^5^, *p* ≤ 0.001). The maximum diagnosis rates of HCV among men were recorded in the age group 45–54 years in urban areas in the year 2015 (21.81 per 10^5^) and in rural areas in the year 2016 (11.31 per 10^5^). Among women, maximum values were observed in the age group 55–64 years in urban areas in the year 2017 (19.96 per 10^5^) and in rural areas in the year 2015 (13.57 per 10^5^).

The mortality rates due to HCV were higher in urban than in rural areas (Table 1). There were significant differences in the median HCV mortality rates based on place of residence, except for chronic mortality rates in men aged ≤44 years and total acute mortality rates in women. The total ASMR of HCV in urban men were three times higher than in rural men (0.76 per 10^5^ vs. 0.25 per 10^5^; *p* ≤ 0.001), and in women, they were two times higher in urban compared to rural areas (0.21 per 10^5^ vs. 0.10 per 10^5^; *p* ≤ 0.05), respectively. The ASMR value for acute HCV was low, and in urban areas it was 0.018 per 10^5^ in men and 0.003 per 10^5^ in women; however, in rural areas, the value was about 0 and the data were very unstable. The ASMR values for chronic mortality due to HCV were similar to the values of total mortality due to HCV. The highest values of ASMR for chronic mortality due to HCV were found in the age group 45–64 years, where for men in urban and rural areas, it was 0.31 per 10^5^ vs. 0.15 per 10^5^ (*p* ≤ 0.001), and for women, it was 0.13 per 10^5^ vs. 0.06 per 10^5^ (*p* ≤ 0.002), respectively.

**Figure 1:**
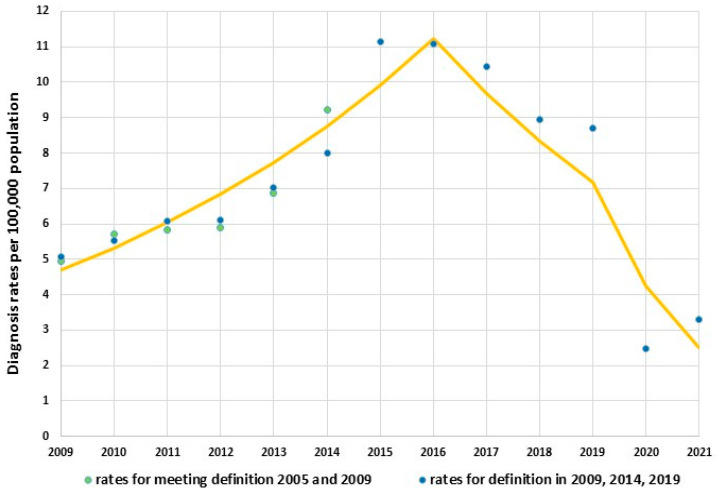
Trends of hepatitis C virus infection in Poland over 2009–2021.

The analysis indicates a significant fluctuation in trends for total diagnosis rate of HCV from 2009 to 2021 (Figure 1). In the initial period of 2009 to 2016, a significant increase in the total diagnosis rate was observed (from 5.08 per 10^5^ to 11.09 per 10^5^) and APC_2009–2016_ was +13.2, 95% CI: 8.0, 18.8, *P*_trend_ < 0.05. From 2016 to 2019, a change in direction of the trend was observed, and diagnosis rate decreased to 8.71 per 10^5^. However, it was not a significant change, and APC_2016–2019_ was −13.9, 95% CI: −39.6, +22.9, *P*_trend_ > 0.05. Sharp reduction in total HCV diagnosis rate was seen after the year 2019 and was 3.26 per 10^5^ in the year 2021; in this period, APC_2019–2021_ was −41.0, 95% CI: −58.7, −15.8, *P*_trend_ < 0.05. In general, these changes in direction of the trend for total diagnosis rate of HCV from positive to negative resulted in the lack of significance of the linear trend throughout the period, in which AAPC_2009–2021_ was −1.6, 95% CI: −8.9, +6.3, *P*_trend_ > 0.05.

In Figure 1 the values of total diagnosis rate of HCV over the years 2009 to 2021 are shown, considering the value of coefficients based on the appropriate definition of HCV. It is worth noting that in most years of the period between 2009 and 2014, the coefficients for these definitions were very similar.

**Figure 2:**
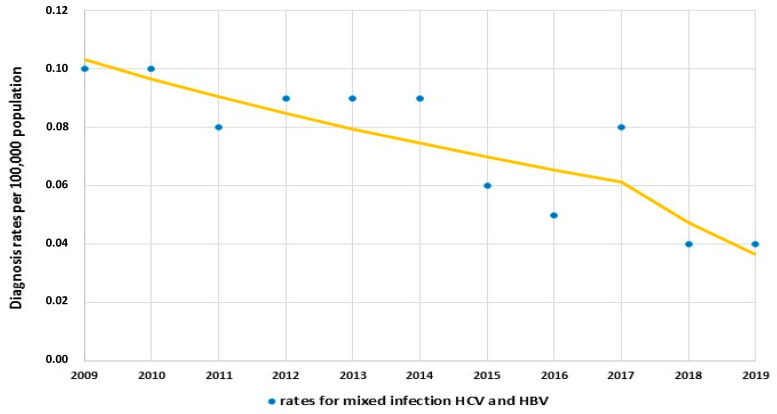
Trends of mixed infection of hepatitis C virus and hepatitis B virus in Poland over 2009–2019.

There was a significant decrease in mixed infection of HCV and HBV diagnosis rate from 0.1 per 10^5^ to 0.08 per 10^5^ (APC_2009–2017_ −6.3, 95% CI: −11.7, −0.5, *P*_trend_ < 0.05). In subsequent years until 2019, the trend still decreased to 0.04 per 10^5^ (APC_2017–2019_ −22.7, 95% CI: −55.2, 33.3, but this trend was insignificant *P*_trend_ > 0.05 (Figure 2).

According to the Polish definition, acute cases of HCV accounted for 1.62% of all diagnosed cases of HCV over the period of 2019–2021 (Table 2). In 2019, the percentage of acute cases was higher (1.91%) when compared to the years 2020 and 2021 (when the COVID-19 pandemic occurred) with percentages of 1.05 and 1.29%, respectively. Between the years 2019 and 2020, the total diagnosis rate of HCV sharply decreased by 71.4% (chronic by 71.2% and acute by 82.3%). During the next year of the pandemic (2021 vs. 2020) rate of HCV increased (chronic by 30.9% and acute by 33.3%).

Table 3 shows changes in the HCV pattern during the COVID-19 pandemic, when the population was under social isolation in 2020. This contributed to a significant reduction in the diagnosis rate of HCV, when compared to the year 2019. The most significant changes were seen among women in the age group ≤ 14 in rural areas, where coefficient decreased by 93.8%. Generally, pronounced changes were observed among young adults in the age group 15–24, in rural (among men by −80.0%, and in women by −90.6%) and urban areas (in men by −84.0%). Among women, in urban areas, in the age group 15–24, the change was by −70.0%; however, the most significant decrease was in the oldest age group ≥ 65 years (by −75.9%). For the whole population, an average decrease in diagnosis rates of HCV in 2020 when compared to 2019 was about 70%; in rural men by 71.4% and rural women by 67.7%, and in urban settings 71.8 and 72.2% for men and women, respectively.

**Figure 3:**
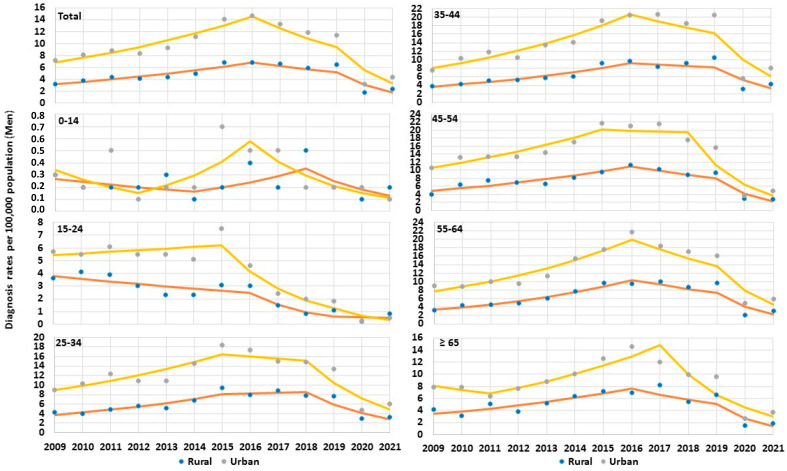
Trends of hepatitis C virus infection among men by age groups in Poland over 2009–2021.

**Figure 4:**
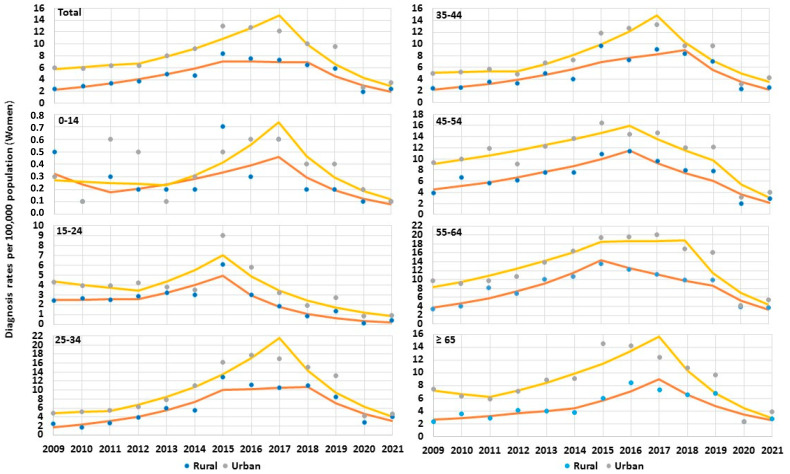
Trends of hepatitis C virus infection among women by age groups in Poland over 2009–2021.

Joinpoint analysis to identify changes in trends of HCV diagnosis rates are presented in Table 4, Figure 3 and Figure 4. The results from the analysis indicate changes in trend direction of HCV during 2009 to 2021, when they appeared in two joinpoints connecting a three-line segment of trend. Among men, in the first period of 2009–2016 (trend 1), an increase in total diagnosis rate of HCV in rural areas from 3.2 per 10^5^ to 6.8 per 10^5^ (APC_2009–2016_ +11.50%, *P*_trend_ < 0.05) and urban areas from 7.1 per 10^5^ to 14.6 per 10^5^ (APC_2009–2016_ +11.44%, *P*_trend_ < 0.05) was observed. In the subsequent years between 2016 and 2019 (trend 2), the reduction in total HCV diagnosis rate was shown among men in rural areas to be 6.4 per 10^5^ (APC_2016–2019_ −8.66%, *P*_trend_ > 0.05) and in urban areas to be 11.4 per 10^5^ (APC_2016–2019_ −13.63%, *P*_trend_ > 0.05). Marked decrease in total HCV diagnosis rate was visible after the year 2019, and in the year 2021, among men in rural areas the rate decreased to 2.3 per 10^5^ (APC_2019–2021_ −41.47%, *P*_trend_ < 0.05), and in urban areas it decreased up to 4.3 per 10^5^ (APC_2019–2021_ − 40.88%, *P*_trend_ < 0.05). In the analysis by age, in the age groups ≤ 14, 15–24, 25–34 among men in rural areas over 2009–2021 none of the three-line segments was significant (*P*_trend_ > 0.05). However, in the following age groups 35–44, 45–54, and 55–64 among men in rural areas in the initial period of 2009–2016 (trend 1), HCV diagnosis rate increased (*P*_trend_ < 0.05); the second line segment showed no significant changes (*P*_trend_ > 0.05), and after 2019, there was a sharp diminishing trend (*P*_trend_ < 0.05). In the oldest age group ≥ 65 years among rural men only trend 1 was increasing (*P*_trend_ < 0.05), and there were no significant changes in subsequent years. Among urban men, the first line segment of HCV diagnosis rate was rising in age groups 35–44 (*P*_trend_ < 0.05) and 55–64 (*P*_trend_ < 0.05) over 2009–2016. The second line segment decreased only in the period of 2015–2019 in the age group 15–24 (*P*_trend_ < 0.05); the third line segment showed significant decline in most of age groups, with the exception of the age groups 25–34 and 45–54 (Table 4 and Figure 3).

Among women, changes in the direction of trends of HCV diagnosis rates occurred; however, they were not pronounced as in the case of men. In rural areas, the first line segment of total diagnosis rate of HCV among women was rising in the period of 2009–2015 from 2.4 per 10^5^ to 8.3 per 10^5^ (APC_2009–2015_ +20.53%, *P*_trend_ < 0.05), in subsequent years (trends 2 and 3), the rate decreased, but these changes were not statistically significant (*P*_trend_ > 0.05). In urban areas, among women significant trend changes appeared later in the years 2017–2021 and total diagnosis rate of HCV decreased from 12.2 per 10^5^ to 3.5 per 10^5^ (APC_2017–2021_ −33.58%, *P*_trend_ < 0.05). Considering age groups, in rural areas among women in the age groups 25–34, 35–44, 45–54, 55–64, the first line segment of HCV diagnosis rate in the years 2009–2015/2016 significantly increased (*P*_trend_ < 0.05). The second trend in age groups 25–34, 35–44, 45–54, 55–64 was insignificant (*P*_trend_ < 0.05), and third trend was negative, but significantly decreased in age groups 35–44 and 55–64 (*P*_trend_ < 0.05). In the oldest age group ≥ 65, until 2018 the changes were insignificant and after this period the trend 3 for diagnosis rate of HCV decreased significantly (*P*_trend_ < 0.05). In urban women, the first line segment showed an increase only in the age group 45–54 during 2009–2016 (*P*_trend_ < 0.05). The second line segment increased only in the age group 25–34 in 2011–2017. The third line segment showed a significant decline in the age group 15–24 over 2015–2021, in age groups 25–34 and 35–44 over 2017–2021, and in the age group 45–54 over 2019–2021 (Table 4 and Figure 4).

Significant changes in the diagnosis rate of HCV occurred in the age group 15–24 years in rural and urban areas in both genders during the period of 2009–2021. In this age group there were decreasing trends among men in rural areas (AAPC_2009–2021_ −16.92%, *P*_trend_ < 0.05), and urban areas (AAPC_2009–2021_ −19.21%, *P*_trend_ < 0.05). Similarly, a decreasing trend was noted among women in rural areas (AAPC_2009–2021_ −17.30%, *P*_trend_ < 0.05) and urban areas (AAPC_2009–2021_ −10.89%, *P*_trend_ < 0.05). Over 2009–2021 in the age groups ≤ 14 years and ≥25 years, the trends were insignificant (*P*_trend_ > 0.05).

Throughout the analyzed period of 2009–2021, there were statistically significant differences in linear trends of total HCV diagnosis rate in men of urban (AAPC −2.48%) and rural areas (AAPC −0.94%) in *p* = 0.007, and in the age group 25–34 years (AAPC in urban −2.32% and rural +0.56%, *p* = 0.004). Among women, there occurred significant differences in linear trends for HCV diagnosis in total (AAPC in urban −1.66% and rural +1.66%, *p* = 0.004) and in the age group ≥ 65 years (AAPC in urban −2.48% and rural +3.29%, *p* = 0.004). Among women, the decrease in value of HCV diagnosis rate in the age group of 15–24 years was faster in rural areas (AAPC −17.30%) when compared to urban areas (AAPC −10.88%), *p* = 0.001; whereas in the age group 25–34 years, HCV diagnosis rate increased faster in rural areas (AAPC +8.72%) when compared to urban areas (AAPC +3.80%), *p* = 0.020 (Table 4).

The analysis of mortality caused by HCV between 2009 and 2021 shows a change in the trend direction, with one joinpoint connecting two line segments of the trend (Table 5). The trend of total ASMR due to HCV was more significant among men compared to women, and the trend direction changed from a positive trend 1 (*P*_trend_ > 0.05) to a negative trend 2 (*P*_trend_ < 0.05) around the turn of 2014/2015. Among men in rural areas, a significant decrease in total ASMR due to HCV from 0.47 per 10^5^ to 0.12 per 10^5^ was observed (APC_2014–2021_ −17.17%). A similar decrease was observed among urban men, from 0.95 per 10^5^ to 0.26 per 10^5^ (APC_2015–2021_ −21.55%). Among urban men, there was a significant shift in the trend direction of chronic ASMR due to HCV, which increased from 0.61 per 10^5^ to 0.94 per 10^5^ (APC_2014–2021_ +7.47%), but subsequently decreased to 0.25 per 10^5^ APC_2014–2021_ −21.83%) until 2021. In rural areas, chronic ASMR due to HCV decreased from 0.43 per 10^5^ to 0.12 per 10^5^ (APC_2014–2021_ −16.83%, *P*_trend_ < 0.05) after 2014.

Among women, in rural areas, the trends of total and chronic ASMR due to HCV over the study period were not significant (*P*_trend_ > 0.05), but the trend changed direction to negative in 2013. In urban areas, a significant decreasing trend 2 of chronic ASMR due to HCV from 0.27 per 10^5^ to 0.11 per 10^5^ (APC_2015–2021_ −16.50%) was observed among women.

The study found that significant changes in mortality due to HCV were only observed in urban areas, where the trends were decreasing throughout the period of 2009–2021. In urban areas among men, the decrease was significant for both total ASMR due to HCV (AAPC_2009–2021_ was −8.43%, *P*_trend_ < 0.05) and acute ASMR due to HCV (AAPC_2009–2021_ was −8.34%, *P*_trend_ < 0.05). Similarly, in urban areas among women, the changes were significant for total mortality (AAPC_2009–2021_ was −6.57%, *P*_trend_ < 0.05) and chronic mortality (AAPC_2009–2021_ was −6.64%, *P*_trend_ < 0.05).

The study did not find statistically significant differences in linear trends of total and chronic ASMR due to HCV by place of residence in both genders (*p* > 0.05). This suggests that the overall dynamics of ASMR trend were similar in urban areas compared to rural areas.

## 4. Discussion

In our study on HCV epidemiology in Poland during a 13-year period, we found changes in the trend direction for the diagnosis and mortality rates of HCV. Fluctuations in the number of recorded HCV infections over the analyzed period could be related to changes in the way epidemiological surveillance of HCV was conducted, including changes in the case definitions used. It should be emphasized that changes in the approved HCV definition resulted from the implementation of EU directives regarding changes in disease definitions. In addition, there were legislative changes at the national level that affected the tightening of the HCV case reporting system [23,28]. Treatment using modern medical technologies, such as DAA, contributed to the changes in the HCV trend, but the COVID-19 pandemic had an exceptional impact [16,29]. Our study showed that the occurrence of positive or negative trends in the diagnosis rate of HCV coincided with the directions of the trends in mortality due to HCV, which validates the obtained results. Moreover, our findings are similar to those reported by studies conducted in other countries [29,30,31,32].

The trend diagnosis rate of HCV over 2009–2016 successively increased regardless of the adopted definitions in this period. This was a change from the previous definition from 2005, which included clinical symptoms, and since 2009, all laboratory-confirmed cases have been recorded [22]. Legislative changes played a role in this increase, such as the introduction of a program of routine screening of pregnant women for HCV in 2011 and decisions increasing the sensitivity of supervision in 2014 [28]. This is evidenced by the growth of an upward trend 1 in women aged 25–44 years in rural areas, which was visible during 2009–2015. On the other hand, the introduction of screening tests for pregnant women appeared as an upward trend 2 in urban areas in women aged 15–44 at the turn from 2011 to 2012, and in rural areas in the age group aged 15–24 years from 2012. For men, the results of trend 1 were quite consistent between rural and urban areas, and in the period 2009–2015/2016, there was an increase in the age group 35–64; similar changes occurred in women in rural and urban areas in the age group 45–64 years. These trends may be related to the obligation of laboratories to report positive results from 2014. It should also be noted that media campaigns aimed at the entire population in order to raise health awareness and provide free laboratory tests played an important role in the increase in HCV numbers during the period of 2009–2016. Nationwide educational campaigns were also conducted for occupational target groups with an increased risk of transmission of blood-borne infections, as well as local initiatives to prevent HCV infections [28]. The incidence of reported HCV infections increased until 2015 (trend 1), but subsequently decreased from 11.1 to 8.7 in the period 2016–2019 (trend 2) due to the introduction of DAA medications. A publicly funded program for interferon-free therapies was initiated in 2015 with nearly 100% effectiveness [16]. Our study indicates that the shift in mortality trends from positive to negative occurred at the turn of 2013–2015, indicating that patients with HCV had early access to treatment. The multicentered AMBER project involving 209 patients who received therapy showed a 99% success rate [33]. It is noteworthy that during the transition from the interferon to non-interferon era from 2013 to 2016, the effectiveness of treatment increased from 47 to 98%, as observed in the multicentered EpiTer-1 study involving 9800 patients [34]. These improved therapeutic options resulted in increased HCV recoveries, leading to a reduction in mortality. In the period 2016–2019 (trend 2), the negative value of the trend was observed, particularly in men, with the fastest decline in the 15–24 age group. This suggests that young men, who often engage in risky behaviors related to drug injection, benefited significantly from the implementation of DAA medications.

The COVID-19 pandemic has caused a sharp decrease in recorded cases of infectious diseases, including HCV. In Poland, the situation was unfavorable compared to other EU countries [35]. This was due to numerous restrictions in specialist medical care, including access to diagnostic testing and drug programs. In addition, medical staff was shifted to care for COVID-19 patients [29]. COVID-19 has halted most of the screening activities in Poland. This was due to the fact that almost all centers treating HCV infections were entirely dedicated to COVID-19. As a result, the number of diagnosed people not only decreased, but also the access to therapy was difficult, and sometimes impossible. In addition, many patients, despite the diagnosis of HCV infection, were afraid to seek help from health care institutions. As a result, in 2020 the number of patients treated decreased by 63% compared to 2019, and this decrease continued until 2022 [15]. It is unlikely that the number of HCV infections will actually decrease, because currently there are not many cases that were diagnosed long ago and are identified as newly diagnosed. The trend 3 observed in the period 2018/2019–2021 showed a clear decrease in men, in most age groups. In women, the negative trends usually began in 2017/2018, indicating that the decrease in the diagnosis rate was associated not only with the COVID pandemic but also with the effects of DAA use. However, the negative trend 3 in women aged 45–54 was clearly related to the pandemic period.

In the analysis of the initial period of the COVID-19 pandemic (2019–2020), the overall diagnosis rate for HCV dramatically decreased to 71.4%, with chronic cases decreasing to 71.2% and acute cases decreasing to 82.3%. Notably, significant changes were observed among young adults (aged 15–24 years) who experienced social isolation due to distance learning during the pandemic, resulting in limited direct contact. The rate of recorded infections decreased the most among young women in rural areas (90%) compared to urban areas (70%), while young men in both rural and urban areas experienced a similar decrease (80 and 84%, respectively). Only in the 15–24 age group, the AAPC for diagnosis rates was statistically significant in both rural and urban areas and showed a negative value, indicating a large decrease in rates during the pandemic (Table 4). Among young women in rural areas, AAPC decreased significantly faster compared to urban areas (−17.30 vs. −10.89%), suggesting that young women in urban areas are more burdened with other risk factors for HCV infection, possibly related to the accessibility of beautifying and cosmetic procedures that may cause tissue damage.

The results revealed health inequalities related to the place of residence, with significantly lower diagnosis and mortality rates of HCV in rural areas, which is consistent with findings from other studies [36,37]. This could be explained by limitations in accessibility to healthcare services, less developed infrastructure, and a shortage of healthcare staff, including infectious disease specialists. These factors reduce the possibility of diagnosing HCV and extend waiting times for treatment [36,37]. Limitations in access to medical care in rural areas may also be a reason for the less beneficial changes in HCV trends observed in rural areas compared to urban areas, except for AAPC of women aged 15–24, as explained above. Furthermore, limitations in medical care may be related to financial expenditure. The current level of financing for HCV treatment in Poland significantly differs from the level of financing in other EU countries, resulting in reduced access to treatment and delays in elimination programs [38]. Consequently, only 2% of HCV patients were currently treated in Poland, and the lack of a vaccine makes it even more difficult to fight the disease. In addition to place of residence, age and gender are significant factors that contribute to potential inequalities in the burden of HCV. In both urban and rural areas, the rate of diagnosis increases with age, but a significant rise in infections after the age of 44 may indicate greater exposure to risk factors for HCV. Medical procedures such as surgeries, endoscopic procedures, and dental work, as well as hospitalizations, are frequent at this age and are the main source of infections (80%) in Poland [39]. Male gender is a factor associated with a higher diagnosis and mortality rate, especially in urban areas where high-risk behaviors such as injection drug use, homosexual contacts, or multiple sexual partners are more common [40]. Some studies have highlighted that urban and rural environments have different risk levels, particularly among injecting drug users, and health interventions should be appropriately focused on the community [41]. However, it is worth noting that the differences in diagnosis rates between men and women in rural areas were not as clear as those in urban areas. This may indicate that men in rural areas contact medical care to a lesser extent or that infections are detected accidentally before surgical or diagnostic procedures.

Our results suggest that place of residence, age, and gender are crucial in identifying new HCV infections. Knowledge of these factors should be taken into account when planning preventive measures to reduce infection transmission. The problem of HCV in rural areas requires comprehensive initiatives to increase the diagnosis of infections, improve access to medical care, and provide public education. Highly developed countries, such as Germany, have successfully reduced the burden of HCV by implementing screening tests in groups with a potential increased risk of infection, such as health care workers, people using injection drugs, people infected with HIV, people with increased activity of enzymes from the group of aminotransferases, or immigrants [42]. Such activities may be of particular importance in Poland due to the mass influx of refugees from Eastern Europe who may have a high prevalence of HCV infections. Although there is currently no national HCV prevention strategy in Poland, important decisions have been made at the government level regarding the implementation of the HCV eradication program in penitentiary institutions [43]. Health interventions that lead to the diagnosis of HCV infection and the initiation of treatment may have an impact on reducing long-term complications of HCV infection, such as hepatocellular carcinoma and liver cirrhosis [44]. Consequently, this will result in benefits such as lower costs incurred by the medical care system and social costs resulting from reduced productivity and premature withdrawal from the labor market.

## 5. Limitations and Strengths

The study’s findings should be considered in light of certain limitations. Firstly, the data used for the analysis were limited to newly diagnosed cases of HCV infection reported by gender, age, and place of residence through routine epidemiological surveillance, which may underestimate the scale of infections in Poland due to limited access to medical care and HCV testing [45]. Moreover, there may be variations in the reliability of HCV infection records by physicians [46,47], and the actual number of new infections may not be accurate. Previous studies have also highlighted problems with the reliability of HCV infection registration in the epidemiological surveillance systems of many countries [13,48]. Similarly, the quality of data on registered deaths may be limited, especially in cases of underdocumented death certificates [49]. Deaths were not always accurately recorded in the description of the chain of causes responsible for death, even in countries with advanced health information systems. For example, in the United States, only 19% of patients who died due to HCV had the infection listed on their death certificate, despite 66% having pre-existing indications of chronic liver disease [49]. Therefore, the data on deaths may be underestimated, and the burden of mortality may be lower. However, the method for classifying deaths was constant over time in this study, so the time trends reported here are likely to be reliable. Secondly, national registers do not take into account information on HCV risk factors, which could contribute to an increase in the diagnosis rate. However, there are insufficient sources to establish risk factors for the analyzed years of 2009–2021. Finally, registries do not provide information on the number of reinfections in recovered people. Thirdly, the study could not assess changes in the time trend separately for acute and chronic diagnosis rates of newly diagnosed HCV infections. The epidemiological surveillance system in Poland defined acute and chronic HCV cases only in 2019 [22], and the study, which covered 13 years of follow-up, did not have adequate data to differentiate new HCV cases between acute and chronic.

Despite potential limitations, this study offers significant advantages, including the use of complete national data on HCV cases covering the entire Polish population. As a result, the analysis provides an important source of evidence for assessing population health. By examining HCV epidemiology, our study reveals health inequalities related to socio-demographic factors such as place of residence, gender, and age. Moreover, this study is unique in that it represents the first long-term analysis of HCV trends using joinpoint regression analysis, with a particular focus on mortality rates [30,48]. This method allowed us to not only estimate trends and identify significant changes over time but also to demonstrate differences in the dynamics of trends between urban and rural areas. In light of these findings, our study could be used to monitor changes in HCV infections and deaths and to guide future efforts to reduce the burden of this disease.

## 6. Conclusions

This population study conducted in Poland indicates that the trend of HCV infections changed direction during 2009–2021. The increase in recorded HCV infections was reversed at the turn of 2015–2016, and a sharp decrease in the trend was observed during the COVID-19 pandemic. This could present a challenge for healthcare systems in the coming years, and it is therefore crucial to continue monitoring the impact of the pandemic on the viral hepatitis response. Health inequalities by place of residence, with lower levels of HCV diagnosis and mortality observed in rural areas, highlight the need for financial investments by the state for screening and the use of diagnostic tests, and should be included as an important direction for future research. To combat HCV, it is important to intensify screening by launching national HCV eradication programs for both the entire population and at-risk groups, with a particular emphasis on prison inmates who have the highest infection rates. Additionally, it is vital to improve the linkage to care, so that diagnosed patients receive treatment as soon as possible.

## Figures and Tables

**Table 1:** Distribution of descriptive statistics for HCV infection by gender, place of residence, and age over the years 2009–2021.

Age Group	Rural Areas			Urban Areas			* *p*
	Me	Min	Max	Me	Min	Max	
	Diagnosis Rates of HCV per 100,000 Population						
Men							
Total	4.34	1.84	6.80	9.29	3.23	14.63	0.001
≤14	0.23	0.08	0.46	0.24	0.06	0.72	0.249
15–24	2.32	0.22	4.09	5.11	0.29	7.53	0.002
25–34	5.56	2.85	9.41	12.36	4.68	18.29	0.001
35–44	5.85	3.05	10.46	13.41	5.62	20.61	0.001
45–54	7.37	2.70	11.31	14.54	3.57	21.81	0.001
55–64	6.12	2.10	9.88	11.29	4.81	21.70	0.001
≥65	5.08	1.48	8.22	8.85	2.56	14.64	0.001
Women							
Total	4.70	1.91	8.28	7.98	2.63	13.02	0.001
≤14	0.16	0.01	0.74	0.37	0.06	0.57	0.196
15–24	2.49	0.12	6.06	3.85	0.80	9.00	0.001
25–34	5.47	1.56	12.83	7.76	4.17	17.82	0.001
35–44	3.98	2.30	9.63	6.77	3.24	13.29	0.001
45–54	7.49	1.89	11.40	12.02	3.09	16.35	0.001
55–64	9.87	3.38	13.57	13.86	4.19	19.96	0.001
≥65	3.95	2.27	8.37	8.75	2.31	14.53	0.001
	Age-standardized mortality rate of HCV per 100,000 population						
Men							
Total	0.25	0.11	0.47	0.76	0.26	0.98	0.001
Acute HCV (Total)	0.000	0.000	0.031	0.018	0.000	0.065	0.013
Chronic HCV (Total)	0.25	0.11	0.43	0.70	0.27	0.95	0.001
≤44	0.04	0.02	0.10	0.07	0.01	0.10	0.133
45–64	0.15	0.08	0.31	0.31	0.13	0.49	0.001
≥65	0.01	0.003	0.04	0.31	0.04	0.53	0.006
Women							
Total	0.10	0.01	0.18	0.21	0.01	0.34	0.023
Acute HCV (Total)	0.000	0.000	0.018	0.003	0.000	0.027	0.534
Chronic HCV (Total)	0.10	0.01	0.18	0.21	0.01	0.32	0.001
≤44	0.01	0.00	0.03	0.02	0.00	0.06	0.001
45–64	0.06	0.00	0.14	0.13	0.00	0.20	0.002
≥65	0.03	0.00	0.04	0.05	0.002	0.08	0.002

Abbreviations: HCV—hepatitis C virus, Max.—maximum, Me—median, Min.—minimum; * Wilcoxon’s signed-rank test.

**Table 2:** Case classification of acute and chronic HCV infection in Poland over the years 2019–2021.

Years	Acute Cases	Chronic and Unknown Cases	Total
	Number of reported HCV cases *		
2019–2021	90	5452	5542
2019	64	3279 **	3343
2020	10	945	955
2021	16	1228	1244
	Diagnosis rate of HCV per 100,000 population *		
2019	0.17	8.54	8.71
2020	0.03	2.46	2.49
2021	0.04	3.22	3.26

Abbreviations: HCV—hepatitis C virus; * According to the Polish definition of HCV; ** Including 14 cases of mixed HBV and HCV infection.

**Table 3:** Changes in the HCV diagnosis rates * by gender, place of residence, and age group during the COVID-19 pandemic in 2019 and 2020.

	Men			Women		
Calendar Year	2019	2020	% Change 2020/2019	2019	2020	% Change 2020/2019
Age group	Rural areas					
Total	6.43	1.84	−71.4	5.91	1.91	−67.7
≤14	0.23	0.08	−65.2	0.16	0.01	−93.8
15–24	1.10	0.22	−80.0	1.28	0.12	−90.6
25–34	7.63	2.85	−62.6	8.38	2.77	−66.9
35–44	10.46	3.05	−70.8	7.05	2.30	−67.4
45–54	9.44	2.94	−68.9	7.83	1.89	−75.9
55–64	9.57	2.10	−78.1	9.87	3.95	−60.0
≥65	6.62	1.48	−77.6	6.84	2.27	−66.8
	Urban areas					
Total	11.44	3.23	−71.8	9.46	2.63	−72.2
≤14	0.23	0.17	−26.1	0.37	0.18	−51.4
15–24	1.81	0.29	−84.0	2.67	0.80	−70.0
25–34	13.31	4.68	−64.8	13.23	4.17	−68.5
35–44	20.40	5.62	−72.5	9.71	3.24	−66.6
45–54	15.59	3.57	−77.1	12.11	3.09	−74.5
55–64	16.13	4.81	−70.2	15.96	4.19	−73.7
≥65	9.57	2.56	−73.2	9.57	2.31	−75.9

Abbreviations: HCV—hepatitis C virus; * Per 100,000 population.

**Table 4:** Trends in diagnosis rates of HCV by gender, place of residence, and age in Poland over the years 2009–2021 with model-based estimations.

Age Group	Trend 1		Trend 2		Trend 3		Trend 2009–2021	** *p*
	Years	APC	Years	APC	Years	APC	AAPC	
	**MEN**							
	**Rural areas**							
Total	2009–2016	11.50 *	2016–2019	–8.66	2019–2021	−41.47 *	−0.94	**0.007**
≤14	2009–2014	−9.33	2014–2018	20.79	2018–2021	−28.71	−1.40	0.740
15–24	2009–2016	−5.85	2016–2019	−38.04	2019–2021	−9.15	−16.92 *	0.646
25–34	2009–2015	13.62	2015–2018	1.70	2018–2021	−30.39	0.56	**0.004**
35–44	2009–2016	14.05 *	2016–2019	−3.53	2019–2021	−36.70 *	2.81	0.070
45–54	2009–2016	12.20 *	2016–2019	−10.04	2019–2021	−46.94 *	−1.90	0.057
55–64	2009–2016	17.72 *	2016–2019	−10.92	2019–2021	−45.54 *	1.00	0.254
≥65	2009–2016	11.98 *	2016–2019	−12.71	2019–2021	−46.98	−2.88	0.522
	**Urban areas**							
Total	2009–2016	11.44 *	2016–2019	−13.63	2019–2021	−40.88 *	−2.48	
≤14	2009–2012	−23.68	2012–2016	39.56	2016–2021	−28.46 *	−2.94	
15–24	2009–2015	2.29	2015–2019	−33.25 *	2019–2021	−47.56 *	−19.21 *	
25–34	2009–2015	10.76	2015–2018	−2.60	2018–2021	−31.32	−2.32	
35–44	2009–2016	14.35 *	2016–2019	−7.62	2019–2021	−38.84 *	1.36	
45–54	2009–2015	11.18	2015–2018	−1.10	2018–2021	−42.45	−4.66	
55–64	2009–2016	14.66 *	2016–2019	−11.84	2019–2021	−42.32 *	−0.39	
≥65	2009–2011	−7.90	2011–2017	13.74	2017–2021	−33.02 *	−3.58	
	**WOMEN**							
	**Rural areas**							
Total	2009–2015	20.53 *	2015–2018	−0.77	2018–2021	−34.50	1.66	**0.004**
≤14	2009–2011	−26.63	2011–2017	17.38	2017–2021	−35.38	−4.66	0.297
15–24	2009–2012	1.47	2012–2015	24.39	2015–2021	−40.93 *	−17.30 *	**0.001**
25–34	2009–2015	34.94 *	2015–2018	2.41	2018–2021	−34.32	8.72	**0.020**
35–44	2009–2015	21.46 *	2015–2018	8.75	2018–2021	−37.84 *	4.17	0.061
45–54	2009–2016	14.45 *	2016–2019	−19.30	2019–2021	−40.63	−2.77	0.112
55–64	2009–2015	25.20 *	2015–2019	−11.99	2019–2021	−39.03	1.43	0.231
≥65	2009–2014	10.93	2014–2018	26.86	2018–2021	−27.56 *	3.29	**0.004**
	**Urban areas**							
Total	2009–2012	5.09	2012–2017	17.15	2017–2021	−33.58 *	−1.66	
≤14	2009–2013	−3.92	2013–2017	33.60	2017–2021	−36.74	−0.62	
15–24	2009–2012	−7.52	2012–2015	26.86	2015–2021	−30.05 *	−10.89 *	
25–34	2009–2011	4.31	2011–2017	26.24 *	2017–2021	−33.93 *	3.80	
35–44	2009–2012	1.35	2012–2017	22.63	2017–2021	−30.51 *	1.45	
45–54	2009–2016	8.42 *	2016–2019	−15.18	2019–2021	−44.16 *	−5.13	
55–64	2009–2015	14.32	2015–2018	0.54	2018–2021	−38.60	−1.70	
≥65	2009–2011	−7.48	2011–2017	16.77	2017–2021	−34.40	−2.48	

Abbreviations: APC—annual percent change, AAPC—average annual percent change, HCV—hepatitis C virus; * Statistically significant trend at *p* < 0.05; ** Wald test for the significance of differences in trends of HCV diagnosis in total and in the relevant age groups between rural and urban areas in the years 2009–2021.

**Table 5:** Trends in age-standardized mortality rates of HCV by place of residence and gender in Poland in the years 2009–2021 with model-based estimations.

Mortality	Trend 1		Trend 2		Trend 2009–2021	** *p*
	Years	APC	Years	APC	AAPC	
	MEN					
	**Rural areas**					
Total	2009–2014	20.26	2014–2021	−17.17 *	−4.40	0.121
Chronic HCV	2009–2014	19.65	2014–2021	−16.83 *	−4.34	0.125
	**Urban areas**					
Total	2009–2015	6.89	2015–2021	−21.55 *	−8.43 *	
Chronic HCV	2009–2015	7.47 *	2015–2021	−21.83 *	−8.34 *	
	WOMEN					
	**Rural areas**					
Total	2009–2013	6.34	2013–2021	−11.40	−6.84	0.337
Chronic HCV	2009–2013	8.42	2013–2021	−13.94	−8.30	0.639
	**Urban areas**					
Total	2009–2015	2.95	2015–2021	−23.38	−6.57 *	
Chronic HCV	2009–2015	4.37	2015–2021	−16.50 *	−6.64 *	

Abbreviations: APC—annual percent change, AAPC—average annual percent change, HCV—hepatitis C virus. * Statistically significant trend at *p* < 0.05. ** Wald test for the significance of differences in trends of HCV mortality between rural and urban areas in the years 2009–2021.

## Data Availability

Data were collected from publicly archived datasets analyzed or generated during the study and presented in Table 1.
